# Implant-assisted removable prosthetic rehabilitation after distraction osteogenesis in a patient with ameloblastoma recurrence: A case report

**DOI:** 10.1097/MD.0000000000018290

**Published:** 2019-12-10

**Authors:** Jae-Hyun Lee, Sung-Hun Kim, Hyung-In Yoon, In-Sung Luke Yeo, Jung-Suk Han

**Affiliations:** a Department of Prosthodontics, One-Stop Specialty Center, Seoul National University Dental Hospital; b Department of Prosthodontics and Dental Research Institute, School of Dentistry, Seoul National University, Seoul, South Korea.

**Keywords:** Ameloblastoma, Distraction osteogenesis, Implant-assisted removable partial denture, Mandibulotomy, Treatment plan

## Abstract

**Introduction:**

A resected mandibular edentulous ridge resulting from an ameloblastoma and marginal mandibulectomy is a restorative challenge. To maintain oral hygiene, recurrent examinations, and for long-term maintenance, a removable dental prosthesis is preferred to an implant-supported fixed dental prosthesis.

**Patient concerns:**

A 28-year-old Asian man was referred for evaluation of a radiolucent area on the right side of the mandible. The right mandibular area had increasingly enlarged over a period of ≥5 months. Marginal resection and inferior alveolar nerve repositioning of the mandible were performed by oral surgeons, followed by reconstruction of the resected mandible with distraction osteogenesis. After 6 years, the patient presented with swelling of the same area.

**Diagnosis:**

Histopathological examination revealed recurrence of benign ameloblastoma in the mandible. After mass excision of the recurrent benign tumor, dental implants were installed. To aid with recurrent examinations and oral hygiene maintenance, a treatment plan using implant-assisted removable dental prosthesis, instead of a fixed prosthesis, was formulated.

**Interventions:**

The edentulous area was rehabilitated with a tooth- and implant-assisted removable partial denture. Due to the insufficient intermaxillary clearance, the removable prosthesis was designed in such a manner that retention, support, and stability could be ensured by separate components.

**Outcomes:**

The tooth- and implant-assisted removable partial denture showed satisfactory function and esthetics. No complications were observed in the dental prosthesis and supporting tissues during the 3-year follow-up period.

**Conclusion:**

In recurrent ameloblastoma cases, a removable dental prosthesis may be an effective treatment option for oral rehabilitation. The type of denture design used in this study is novel for implant-assisted removable partial denture rehabilitation.

## 1 Introduction

Ameloblastoma is an odontogenic tumor commonly occurring in the mandible, with the potential to arise throughout the mandibular region.^[1]^ Despite having a benign histological pattern, ameloblastomas have a high recurrence rate,^[2]^ attributed to tumor islands which invade adjacent tissues.^[3]^ To prevent recurrence, mandibular resection is indicated for cases of large or multicystic ameloblastomas.^[4]^

Jaw resection often creates unesthetic facial contours and compromises oral functions such as mastication, speech, and deglutition.^[5]^ An implant-supported fixed dental prosthesis may restore masticatory function.^[6]^ However, the absence of attached keratinized gingiva makes oral hygiene of the peri-implant area difficult, leading to inflammation of the soft tissues.^[7]^ Additionally, the use of fixed dental prosthesis at the site of occurrence complicates the subsequent detection of ameloblastoma recurrence. Thus, removable dental prostheses are recommended. However, the inadequacy of hard and soft tissue at the mandibular resection site presents a challenge for a good removable partial denture with adequate retention, stability, and support.^[8]^

Although implant-supported prosthetic rehabilitation in patients with resected mandibles has been reported,^[9–13]^ few studies have discussed the considerations for restoring resected jaws with removable dental prostheses in patients with limited interocclusal space. This clinical report describes the treatment planning and restoration procedure of a tooth- and implant-assisted removable dental prosthesis in a resected mandible that developed a recurrent ameloblastoma.

### 1.1 Consent statement

The patient has provided informed consent for the publication of this case report and accompanying images.

## 2 Case report

**Figure 1:**
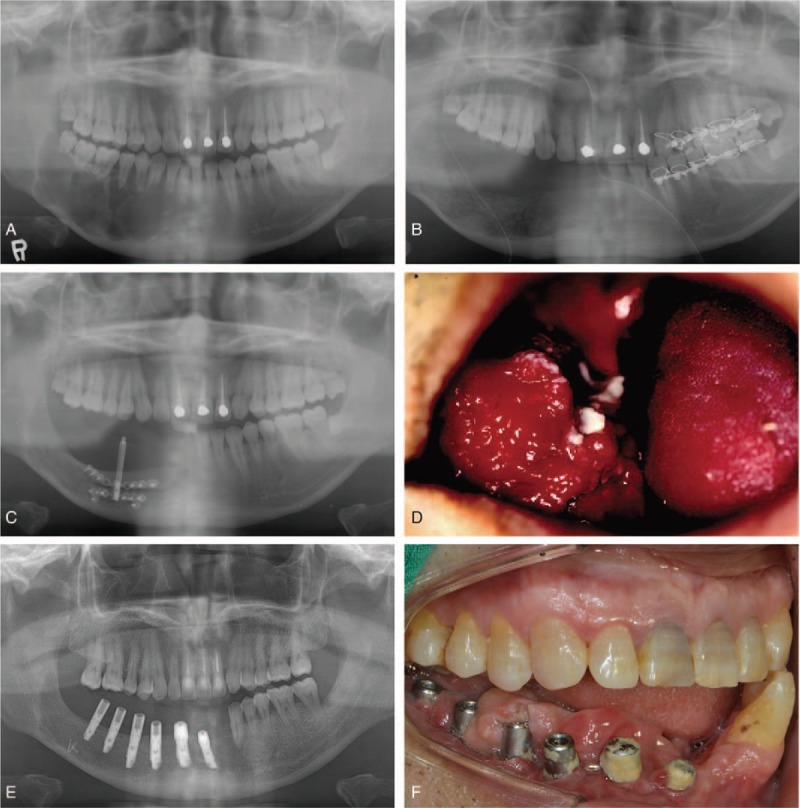
Surgical phase. Panoramic radiographs (A) of the ameloblastoma at the right side of the mandible, (B) of the ameloblastoma after mandibulotomy and nerve repositioning, and (C) of the reconstructed mandible with the distractor. (D) Recurrence of the ameloblastoma. (E) Panoramic radiograph of the mandible after implant placement. (F) Lateral view of the mandible. There was no available space due to the overgrown mucosa. Poor oral hygiene was observed at the healing abutments.

A 28-year-old Asian man presented to the Department of Oral and Maxillofacial Surgery for examination of a radiolucent area on the right side of the mandible in February 2002. The right mandibular area had gradually enlarged over a period of ≥5 months. He had no medical, family, and psychosocial history. Panoramic radiography revealed multilocular radiolucent areas in the mandible, from the symphysis area to the right third molar area (Fig. 1A). Computed tomography revealed a radiolucent area measuring approximately 60 × 30 × 20 mm in size with a well-defined scalloped margin and daughter lesions. Histopathological examination confirmed the diagnosis of benign ameloblastoma.

Marginal mandibulectomy with inferior alveolar nerve repositioning was performed by oral and maxillofacial surgeons. Mandibular reconstruction with a ramal block bone graft was simultaneously performed (Fig. 1B). After 29 months of follow-up, vertical distraction osteogenesis was performed for approximately 3 weeks to increase the bone height of the bone-grafted area. In total, a 10.2-mm height increase was attempted (Fig. 1C). After 5 months, the distractor of the bone-augmented area was removed.

After 6 years, in June 2011, the patient presented with swelling of the same area (Fig. 1D). A biopsy confirmed ameloblastoma recurrence. Mass excision of the recurrent benign tumor was performed.

Implant placement was planned after 4 years of follow-up with no signs of recurrence. Six bone-level dental implants (US II, external hex connection type, Osstem Co., Busan, Korea) were placed. After 3 months, the implants were uncovered, and long titanium cylinders were connected as healing abutments owing to the height of the extremely overgrown, thick soft tissue.

After the second-stage surgery of the implants, the patient was referred to the Department of Prosthodontics for prosthetic rehabilitation (Fig. 1E). At this point, the patient was 42 years old. The surgeon recommended rehabilitation of the edentulous area with a removable dental prosthesis rather than a fixed dental prosthesis, although the implants placed were sufficient for fixed prosthesis. Owing to past recurrence of lesion, periodic examinations of the tissue under the dental prosthesis was advised. Additionally, fixed dental prostheses may present with difficulties in controlling plaque and maintaining oral hygiene due to the lack of keratinized, attached gingiva at the mandibular resection site.

In the edentulous areas where the implants were placed, the soft tissue overgrew, lacking sufficient occlusal clearance with the opposing teeth. There was no unmovable attached gingiva. There was calculus deposition around the healing abutments, and no buccal shelf for the removable partial denture flanges was observed. The mandibular left canine adjacent to the edentulous area was in crossbite with the opposing tooth (Fig. 1F).

**Figure 2:**
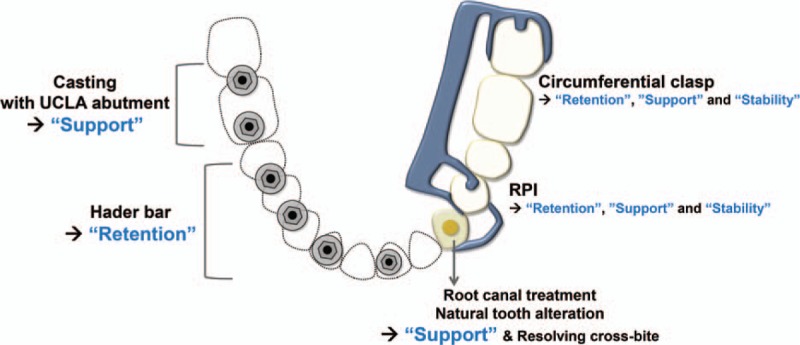
Prosthodontic planning for tooth- and implant-assisted removable partial denture. In cases where-in a milled bar cannot be used, it is necessary to design the removable denture such that retention, support, and stability can be ensured separately.

A tooth- and implant-assisted removable partial denture was planned for oral rehabilitation. On the 2 posterior implants, the dental prostheses lacked space, with inadequate soft and hard tissues to support the denture. Therefore, customization of the abutment to the form of a residual root for exclusively providing support to the removable denture was planned. On the 4 anterior implants, a bar-joint attachment was designed for denture retention. The mandibular left canine, which was in a cross-bite, was to be altered to the residual root-piece form after intentional root canal treatment to provide support for the denture and to establish normal overjet. On the natural mandibular left first premolar and mandibular left second molar, a rest, proximal plate, I-bar (RPI) assembly, and circumferential clasp were designed to provide retention, support, and stability to the denture (Fig. 2).

Natural teeth alterations on the mandibular left canine, first premolar, and second molar were performed with a jig fabricated with acrylic resin (Pattern resin, GC Corp., Tokyo, Japan). The implant-level impression was acquired with the customized tray, pick-up type impression coping and vinyl polysiloxane (Honigum, DMG, Hamburg, Germany). The master cast was fabricated, and bite-jigs were prepared. The intermaxillary jaw relation was recorded with the bite-jigs and rubber-based bite-registration material (Exabite II, GC Corp.).

**Figure 3:**
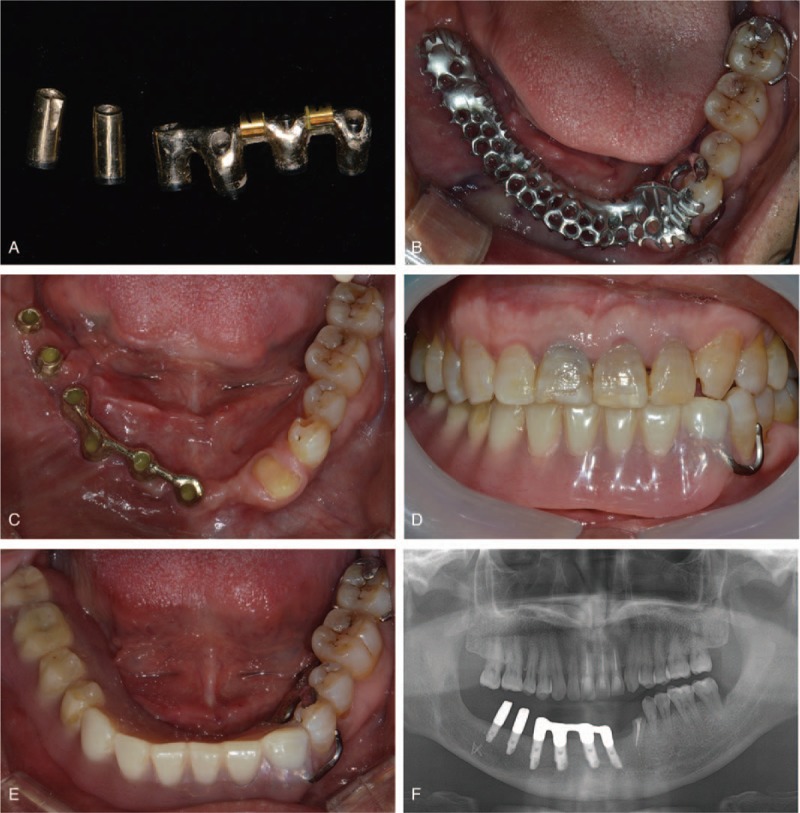
Restorative phase. (A) Casted abutments and bar attachments. (B) Metal framework try-in. (C) Occlusal view of the implant superstructures. (D) Frontal view of the definitive prosthesis. (E) Occlusal view of the implant-assisted removable partial denture (F) Panoramic radiograph at the 3-year follow-up visit.

Six castable UCLA type abutments (UCLA Gold Abutment, Osstem Co.) were used to fabricate attachments on the implants. The 4 abutments for the 4 anterior implants were splinted to each other and attached to the cast gold bar with a plastic Hader bar pattern (Hader Bar, Sterngold, Attleboro, MA). The UCLA type abutments for the 2 posterior implants were shaped to resemble a retained root-piece to support the removable partial denture and cast in gold (Fig. 3A). The casted abutments, bar-attachment, and metal-framework of the removable partial denture were evaluated intraorally, and the fit of the prosthesis was verified (Fig. 3B). The definitive removable partial denture was fabricated using conventional methods. During the delivery appointment, the abutment screws were tightened to 30 Ncm, as per manufacturer's guidelines (Fig. 3C). On follow-up, when the removable partial denture required no further adjustment, the bar attachment clips for the Hader bar were attached using the direct chairside transfer method in the oral cavity with auto-polymerizing acrylic resin (Rebase II, Tokuyama Corp., Tokyo, Japan) (Fig. 3D and E).

During the 3-year follow-up period, the patient could maintain good oral hygiene. No inflammation or ameloblastoma recurrence was observed, and the radiographic marginal bone level around the implants at the site of distraction osteogenesis remained stable (Fig. 3F). No complications occurred in the repositioned inferior alveolar nerve.

## 3 Discussion

Implant-supported fixed partial dentures are often considered the treatment of choice for patients with resected mandibles.^[12]^ Six implants were considered adequate for restoration of the mandibular resected area of the patient in this study with an implant-supported fixed dental prosthesis. However, the patient was provided a removable dental prosthesis to aid recurrent examinations and oral hygiene maintenance.

A milled bar-unit system—providing retention, support, and stability^[9]^ —could not be selected due to insufficient intermaxillary space for a milled bar when referred for restoration to the prosthodontic department. A representative treatment option for fabricating implant-assisted removable dental prostheses in patients with limited interarch space comprises using low-profile solitary attachments such as the Locator system (Zest Anchors, Escondido, CA).^[14]^ However, the Locator system can mainly provide retention and stability to the removable denture.^[15,16]^ Thus, support for the denture should be provided by the underlying soft and hard tissues. However, it is difficult to acquire sufficient denture support from the site of mandibular resection. Therefore, for the patient in this case, it was necessary to design the removable denture in such a manner that retention, support, and stability could be ensured by separate components.

Presently, the 2 posteriorly placed implants could not be splinted because of insufficient intermaxillary space. Instead, the superstructures were fabricated in a root-piece shape to provide denture support without its involvement in denture retention or stability, ensuring that they were not loaded by lateral forces that are unfavorable to the implant fixtures. The 4 anterior implants were splinted and fabricated with a Hader bar attachment to allow denture retention. This type of denture design is novel in implant-assisted partial denture rehabilitation.

The method presented here is limited by a high denture fracture risk and artificial tooth wear.^[17]^ Because the removable prosthesis is supported by dental implants and natural teeth instead of soft tissue, the bite force will be strong^[18,19]^ and can lead to mechanical complications of the dental prosthesis.^[17]^ To prevent wear of the artificial teeth, it may be advantageous to restore the artificial resin teeth with gold crowns or monolithic zirconia crowns. In addition, a periodic recall check-up by dentists is essential for maintenance.

## 4 Conclusion

Tooth- and implant-assisted removable partial dentures can be recommended to provide both functional rehabilitation and oral hygiene maintenance in patients with recurrent ameloblastoma who undergo mandibulotomy and distraction osteogenesis. Depending on the patient's oral condition, a removable dental prosthesis may be a better treatment option than a fixed dental prosthesis.

## Acknowledgments

The authors express their sincere thanks and gratitude to Professor Jong-Ho Lee, Department of Oral and Maxillofacial Surgery, School of Dentistry, Seoul National University, for performing the surgical treatments.

## Author contributions

**Conceptualization:** Jae-Hyun Lee, Sung-Hun Kim, Hyung-In Yoon, In-Sung Luke Yeo, Jung-Suk Han.

**Data curation:** Jae-Hyun Lee.

**Formal analysis:** Jae-Hyun Lee.

**Investigation:** Jae-Hyun Lee.

**Methodology:** Jae-Hyun Lee.

**Project administration:** Jae-Hyun Lee, Sung-Hun Kim, Jung-Suk Han.

**Software:** Jae-Hyun Lee.

**Supervision:** Sung-Hun Kim, Jung-Suk Han.

**Validation:** Hyung-In Yoon, In-Sung Luke Yeo.

**Visualization:** Jae-Hyun Lee.

**Writing – original draft:** Jae-Hyun Lee.

**Writing – review & editing:** Jae-Hyun Lee, Sung-Hun Kim, Hyung-In Yoon, In-Sung Luke Yeo, Jung-Suk Han.
